# Characterization of antimicrobial resistance among *Proteus mirabilis* isolates from catheter-associated urinary tract infections and non-catheter-associated urinary tract infections in Egypt

**DOI:** 10.1186/s12879-025-11118-8

**Published:** 2025-05-27

**Authors:** Noha A. Hassuna, Dalia N. Kotb, Marina Lami, Soha S. Abdelrahim

**Affiliations:** Department of Medical Microbiology and Immunology, Faculty of Medicine, Minia University, Minia, 61511 Egypt

**Keywords:** *Proteus mirabilis*, Catheter-associated, Urinary tract infection, Multidrug resistance, Beta-lactamases, Integrons

## Abstract

**Background:**

Recent worldwide reports of increased numbers of multidrug-resistant (MDR) *Proteus mirabilis* (*P. mirabilis*) isolates, particularly those producing extended-spectrum β-lactamases (ESBLs), are alarming. *P. mirabilis* is a common causative agent of complicated urinary tract infections (UTIs), particularly in patients with long-term urinary catheterization. This study aimed to assess the prevalence, antibiotic resistance patterns, and determinants of *P. mirabilis* among catheter-associated UTIs (CAUTI) and non-catheter-associated UTIs (non-CAUTI).

**Methods:**

One hundred and three *Proteus* strains isolated from 613 UTI patients in Minia, Egypt, were examined for antibiotic resistance patterns, ESBL production, and sulphonamide resistance phenotypically. Class 1 and 2 integrons, ESBL, and *sul* resistance genes were detected by Polymerase chain reaction (PCR), followed by molecular typing of ESBL-producing isolates from catheterized UTI patients using ERIC-PCR.

**Results:**

*Proteus* isolates were detected in 20% of the UTIs, with a higher rate among inpatients (27.3%) compared to outpatients (10.6%). *Proteus* was more significantly isolated from catheterized UTI patients (28.2%, 55/195) than from non-catheterized patients (14.9%, 48/321). Of the 103 *Proteus* isolates, 99 (96.1%) were identified as *P. mirabilis*. High resistance was observed against trimethoprim/sulfamethoxazole (SXT) (80.6%), amoxicillin-clavulanic (AMC) (57.3%), ceftazidime (55.3%), and imipenem (46.6%) antibiotics. Significantly higher resistance rates were observed among *Proteus* isolates from inpatients and catheterized patients. Of the 103 *Proteus* strains, 81 (78.6%) were MDR, with 70.9% of the isolates from catheterized patients. About 74.6% of the isolates from inpatients were MDR. *Sul* genes were detected in 77 isolates (74.7%). The frequency of ESBL-producing *Proteus* isolates was 37.9% which was significantly higher in catheterized patients with increasing dissemination of *bla*_TEM_ genes and *bla*_CTX-M_ genes. *Int1 and Int2* genes were detected in 92.2% and 68.9% of isolates, respectively. ERIC-PCR revealed moderate similarity (65%) between ESBL-producing *Proteus* isolates from catheterized patients.

**Conclusion:**

The high frequency of MDR *P. mirabilis* strains isolated from UTIs in Egypt, particularly among catheterized patients, is a major concern, especially with disseminating class 1 and 2 integrons among isolates. The study also highlights the decreased susceptibility to sulphonamides, 3rd generation cephalosporins, and imipenem, commonly used to treat UTIs. Increased dissemination of ESBL-producing *Proteus* isolates among CAUTIs complicates their treatments. This important pathogen deserves more attention in the future for a better understanding of resistance mechanisms and the dissemination potential of resistant strains.

**Supplementary Information:**

The online version contains supplementary material available at 10.1186/s12879-025-11118-8.

## Background

*Proteus* species are gram-negative members of the *Morganellaceae* family, which forms, together with *Enterobacteriaceae,* the order *Enterobacteriale* [1]. *Proteus mirabilis* (*P. mirabilis*) and *Proteus vulgaris* (*P. vulgaris*) were the most frequent opportunistic pathogenic species of *Proteus* in humans. They widely exist in the environment and the gut microbiota of humans and animals [2]. Urinary tract infection (UTI) is a common health problem in communities and hospitals. Every year, almost 150 million individuals throughout the globe suffer from UTIs, making it a serious public health issue [3].* P. mirabilis* is responsible for 90% of diverse *Proteus* infections, especially UTIs. *P. mirabilis* is a common causative agent of complicated UTIs, particularly in patients with long-term urinary catheterization [4].

Catheter-associated urinary tract infection (CAUTI) is the most common healthcare-associated infection that can cause secondary bloodstream infections, which makes it a severe healthcare burden. *P. mirabilis* produces a urease enzyme that causes hydrolysis of urea, leading to an increase in the urinary pH, thus generating calcium crystals and magnesium ammonium phosphate precipitates. Together, this leads to the formation of crystalline biofilms on catheters [5]**.** According to guidelines published by the Centre for Disease Control and Prevention (CDC); CAUTI is diagnosed if the patient has an indwelling urinary catheter for more than 2 days in the hospital setting and one sign or symptom including fever, suprapubic tenderness, costovertebral angle tenderness in addition to urine culture with more than 10^5^ CFU/mL of one bacterial species [6].

Antibiotic-resistant bacteria are an escalating global health threat. Potent antibiotics are now less effective due to bacterial evolution and gene exchange [7]. *Proteus* isolates that were once largely susceptible to standard antibiotics are now increasingly resistant to UTI treatments. Recent global studies confirm this increasing resistance trend [8–10]. ESBL-producing bacteria are a growing health concern. Recently, the *bla*_CTX-M_ genotype has emerged, replacing *bla*_TEM_ and *bla*_SHV_ in Enterobacteriaceae isolates [11].

Sulfonamides are a group of chemotherapeutic agents that act as competitive inhibitors of the *folP* gene encoding dihydropteroate synthase enzyme (DHPS). The plasmid- and integron-borne *sul1*-*3* genes cause widespread sulfonamide resistance through coding for mutant DHPS enzymes that do not bind to sulfonamides [12]. Trimethoprim/sulfamethoxazole (SXT) is used commonly in developing countries as an empiric treatment of UTI as it is very cost-affordable. ESBLs have been associated with resistance to other antibiotic classes, such as trimethoprim/sulfamethoxazole [13].

Integrons are natural recombination and expression systems that can acquire genes in the form of gene cassettes. In the past two decades, the incidence of integrons in enteric bacteria has increased dramatically with the evolution of multiple gene cassettes, novel gene arrangements, and complex chromosomal integrons [14]. Class 1 and 2 integrons have been associated with antibiotic resistance in *Enterobacteriaceae* such as beta-lactam resistance and sulphonamide resistance, class 2 integrons have been reported with lower prevalence in gram-negative bacteria than class 1 integrons [15].

Despite the increased reports of resistance among *Proteus* strains in the last decade, few studies have addressed this issue in Egypt. In this study, we aimed to determine antimicrobial resistance patterns among *Proteus* isolates obtained from CAUTI and non-CAUTI patients admitted to Minia University Hospitals, Egypt. Then molecular screening of resistant isolates for ESBL, and sulfonamide genes (*sul* genes) was also carried out. All isolates were screened for class 1 and 2 integrons (*int*I1, *int*I2 genes).

## Methods

### Study design

This cross-sectional study included 613 urine samples collected from 336 inpatients and 277 outpatients presenting with UTI symptoms from different departments in Minia University Hospitals. The study was conducted in the Microbiology and Immunology Department, Faculty of Medicine, Minia University, between April 2022 and June 2023. The recruited patients met the following inclusion criteria: adults ≥ 18 years, had no history of receiving antibiotics 48 h before sample collection, patients had one or more of the UTI symptoms (fever, frequent urination, dysuria, incomplete bladder emptying, suprapubic or flank pain). Samples collected from inpatients were obtained 48 h after admission (UTI symptoms appeared 48 h after admission). Demographic data and relevant clinical history data were also collected, including history of catheterization and no history of administration of antimicrobial agents. Informed consent was obtained from all study participants. The Research Ethics Committee of the Faculty of Medicine of Minia University had approved the study protocol with Approval No.276:2/2022.

### Bacterial isolation and identification

Midstream clean-catch urine samples were collected under complete aseptic precautions in sterile screw-capped containers. From catheterized patients, urine was aspirated from the catheter tube after cleaning it with alcohol and puncturing it using a sterile syringe and needle. All samples were transported within 2 h to the microbiology laboratory for immediate examination. Urine samples with pus cells > 10/HPF were streaked on chromogenic media (CHROMagar™ Orientation, Paris, France) by calibrated loop technique [16] Positive cultures with more than 10^5^ CFU/mL colonies were initially identified based on characteristic growth on nutrient agar and MacConkey’s agar media (Oxoid, UK). *Proteus mirabilis* isolates *were* identified phenotypically based on the following tests*:* Oxidase negative, Catalase positive, Indole negative, Methyl red positive, Voger Proskauer negative, Citrate positive, H_2_S production, sugar fermentation of glucose and positive motility test [17]. A total of 103 non-repetitive *proteus* isolates were recovered from all urine samples. Identified isolates were stored by mixing incubated Trypticase soy broth (Oxoid, UK) with sterilized glycerol 20% at −20 ⁰C for further molecular testing.

### Antibiotic susceptibility testing

The antimicrobial susceptibility testing was performed using the Kirby-Bauer disc diffusion technique using Muller-Hinton agar (Oxoid, U.K) according to Clinical Laboratory Standard Institute (CLSI) guidelines, 2023 [18]. The following 11 commercially available discs were used: Amoxicillin/clavulanic acid (AMC) 20/10μg, Ceftriaxone (CRO) 30μg, Ceftazidime (CAZ) 30μg, Imipenem (IPM) 10μg, Amikacin (AK) 30μg, Trimethoprim-Sulfamethoxazole (SXT) 25μg, Cefotaxime (CTX) 30μg, Ciprofloxacin (CIP) 5μg, Cefoxitin (FOX) 30μg, Aztreonam (ATM) 30μg (Oxoid, UK), and Gentamicin (CN) 10µg (Thermo Scientific™, UK). *Escherichia coli* (ATCC 25922) was used as a quality control strain. Isolates that showed resistance to three or more classes of antimicrobial agents have been classified as multidrug-resistant (MDR) [19]. Multiple antibiotic resistance index (MARI) was calculated by the ratio between the sum of antibiotics against which the *proteus* strain displayed resistance and the total number of antibiotics to which the isolate was tested [20].

The minimum inhibitory concentration (MIC) of trimethoprim-sulfamethoxazole (SXT) was evaluated by the broth microdilution method using Muller-Hinton broth (Oxoid, U.K). Ten dilutions of SXT (EIPICO Pharmaceutical Company, Egypt) were tested by the two-fold dilution method in 96 well microtiter plates using a concentration range from 0.25/4.7 µg/ml to 128/2432 µg/ml. According to the CLSI guidelines, MIC ≤ 2/38 µg/mL was considered sensitive and MIC ≥ 4/76 µg/mL was considered resistant to SXT [18].

### ESBL phenotypic confirmation test

Isolates resistant to 3rd generation cephalosporins (ceftazidime zone ≤ 22 mm and cefotaxime zone ≤ 27 mm) were suggested as ESBL producers. The double disc synergy test (DDST) was used to confirm the phenotypic ESBL production according to CLSI guidelines 2023 [18]. Amoxicillin clavulanic acid disk was placed aseptically in the center of the plate containing Mueller–Hinton agar inoculated with standardized inoculum (corresponding to 0.5 McFarland). Then, two test disks of 3rd generation cephalosporins (ceftazidime 30μg and cefotaxime 30μg) were placed at a 15 mm distance from the center of the AMC disk. After overnight incubation at 37 °C, an enhanced zone of inhibition toward AMC (formation of a characteristic keyhole-shaped zone of inhibition between the disks) was considered a synergy between clavulanic acid and any one of the tested antibiotics. This was a phenotypic indication of ESBL production.

### DNA extraction

The genomic DNA of all *Proteus* isolates was extracted using the modified boiling method [21]. Three to five colonies from each isolate were inoculated into tryptone soya broth. Then, 1.5 ml of overnight culture was centrifuged for 5 min at 12,000 rpm at 4 °C. The supernatant was removed carefully, and the pellet was mixed with 200 µl of sterile distilled water. After boiling the suspended pellet for 10 min in a water bath, it was immediately placed in ice for 20 min and then centrifuged at 12,000 rpm for 5 min at 4 °C. The supernatant containing extracted DNA was transferred into a new tube and stored at −20˚C for subsequent PCR amplification.

### Detection of sulfonamide resistance genes

Screening of SXT-resistant isolates of *Proteus* was performed by PCR amplification of *sul1* and *sul2* genes using specific primers to produce PCR products of 347bp and 506bp, respectively; strains positive for *sul1* and *sul2* genes were used as positive controls [22]; (Table 1 s in Supplementary File).

### Detection of ESBL genes

All phenotypic ESBL-producing *Proteus* isolates were screened for the presence of the ESBL genes: *bla*_TEM_*, bla*_SHV_*,* and *bla*_CTX-M 1, 2, 8, 9, 25_ genes. Single PCR reactions were used for the amplification of *bla*_TEM_ and *bla*_SHV_ genes and a multiplex PCR was used to examine the presence of *bla*_CTX-M 1, 2, 8, 9,25_ genes; positive controls were used [23, 24]

### Detection of Class 1 and 2 integron

All isolates were tested for the presence of the conserved regions of class 1 and class 2 integrases [25]. Primer sequences and annealing temperature used for the amplification of *intI*1 and *intI*2 genes were shown in Table 1 s in the Supplementary File).

### Molecular typing by enterobacterial repetitive intergenic consensus‑PCR (ERIC‑PCR)

ESBL-producing* P. mirabilis* isolates from catheterized patients were fingerprinted by ERIC-PCR for the level of genetic similarity using the primer sequences shown in Table 1 s in the Supplementary File. PCR conditions used were as follows: an initial denaturation step at 92 ◦C for 4 min, followed by 40 denaturation cycles of 1 min at 94 ◦C, annealing at 48 ◦C for 1 min, an extension at 72 ◦C for 5 min, and a final extension at 72 ◦C for 5 min [26]. The banding patterns of PCR products were analyzed on a 1.5% agarose gel containing ethidium bromide dye, and the gel was visualized under a UV transilluminator. ERIC-PCR was performed on triplicates to confirm the reproducibility of the amplified band patterns obtained. A similarity dendrogram was constructed by band profile analysis using GelJ software by the unweighted pair group mean method, tolerance value of 1, and Dice's similarity coefficient.

### Statistical analysis

All collected and laboratory data were analysed using statistical analysis by IBM SPSS software (version 19.0). A chi-square test was used for the comparison between groups, and the results were considered significant if the P value was < 0.05.

## Results

### Identification of clinical isolates

**Fig. 1:**
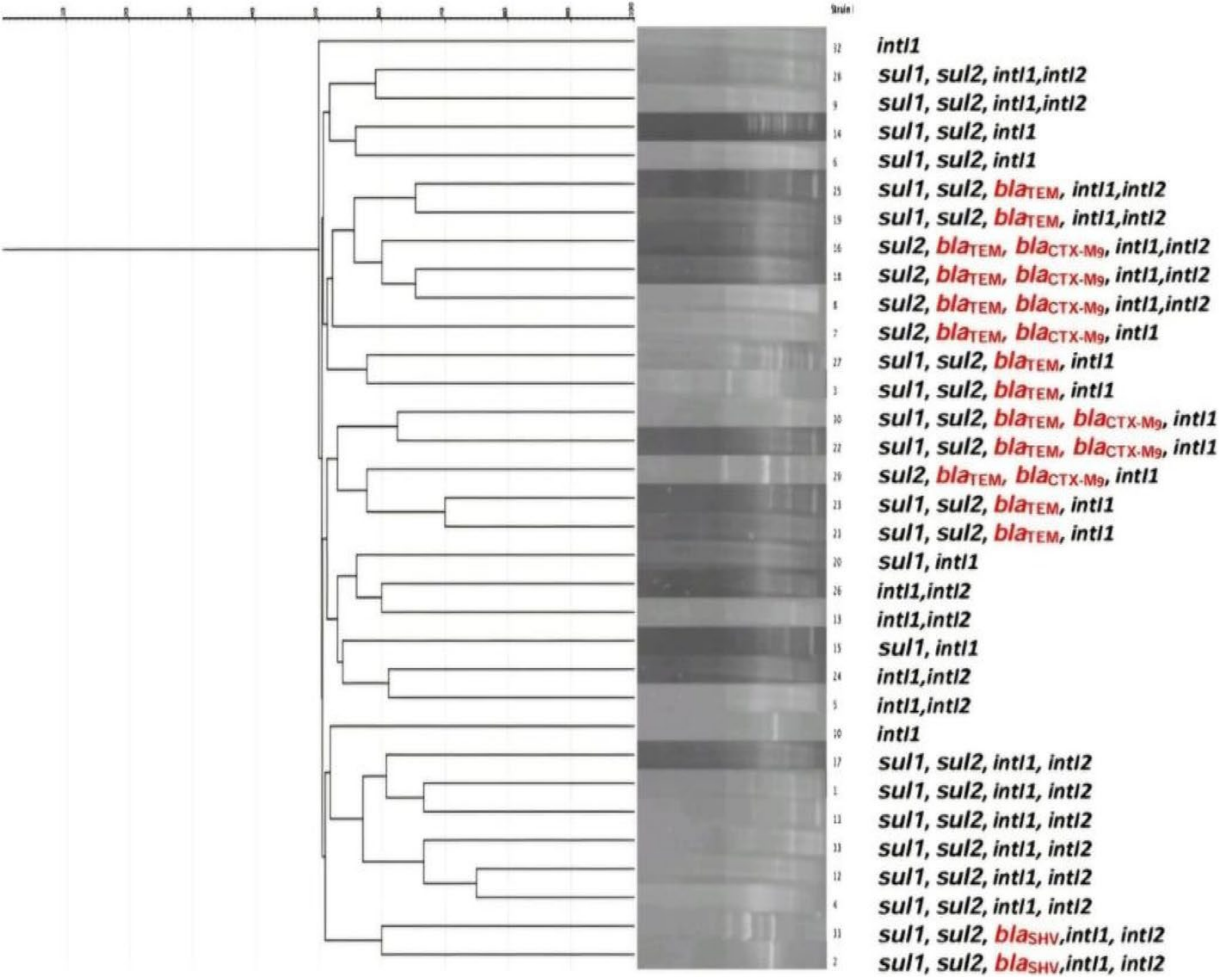
Dendrogram analysis of DNA fingerprinting by ERIC-PCR of ESBL-producing *P. mirabilis* isolates from catheterized patients showing similarity of 65%

Based on the results of urine culture on Chrome agar medium, out of 613 urine samples, 516 (84.2%) of them showed bacteriuria (CFU > 10^5^) while 97 (15.8%) urine samples showed no bacterial growth. Out of 516 UTI patients, *Proteus* species were isolated from 103 samples (20%), while uropathogens other than *Proteus* were isolated from 413 samples (80%) (Fig. 1s Supplementary File). Of the 103 *Proteus* isolates, ninety-nine were identified as *P. mirabilis* (96.1%).

### Demographic characteristics of the study subjects

The prevalence of *Proteus* was higher in catheterized UTI patients (28.2%; 55/195) than in non-catheterized patients (14.9%; 48/321); which was highly significant (P value of < 0.001). The prevalence of *Proteus* among UTI patients was higher in inpatients representing hospital-acquired UTIs (27.3%; 79/289) than in outpatients representing community-acquired UTIs (10.6%; 24/227) which showed high significance (P value of < 0.001). *Proteus* strains were isolated from patients with a mean age of 46.1 ± 15.6 years; 85 (82.5%) of them were adult patients (18–59 years) and 18 (17.5%) aged above 60 years. *Proteus* strains were more predominant among female patients (63/103, 61.2%) than males (40/103, 38.8%). Ten *Proteus* strains (9.7%) were isolated from pregnant women with UTI (Table 2 s Supplementary File).

*Proteus* strains were isolated from patients from different departments (32% from the Gynaecology and Obstetrics Departments, 23.3% from the Urology Department, 21.4% from the ICU, 13.6% from the Surgery Department, and 9.7% from the Internal Medicine Department; Table 1.

**Table 1:** Patient characteristics, clinical data, percentage of antibiotic resistance patterns, and molecular genes among *Proteus* isolates from catheterized and non-catheterized patients

		**Total** **N = 103**	**Catheterized group (*N* = 55)**	**Non-catheterized group(*N*= 48)**	***P*-value**
**Patients'characteristics**					
**Age**	Mean ± SD	46.1 ± 15.6	47.07 ± 15.7	45 ± 15.9	0.511
	Range	18–84	19–84	18–71	
**Gender**	Male	40 (38.8%)	22 (40%)	18 (37.5%)	0.864
	Female	63 (61.2)%	33 (60%)	30 (62.5%)	
**Type of patient**	Outpatient	24 (23.3%)	0 (0%)	24 (50%)	0.000*
	Inpatient	79 (76.7%	55 (100%)	24 (50%)	
**Department**	Gynaecology and obstetrics	33 (32%)	11 (20%)	22 (45.8%)	0.000*
	ICU	22 (21.4%)	22 (40%)	0 (0%)	
	Surgery	14 (13.6%)	10 (18.2%)	4 (8.3%)	
	Internal medicine	10 (9.7%)	4 (7.3%)	6 (12.5%)	
	Urology	24 (23.3%)	8 (14.5%)	16 (33.3%)	
**Dysuria**	Yes	60 (58.3%)	14 (25.5%)	46 (95.8%)	0.000*
	No	43 (41.7%)	41 (74.5%)	2 (4.2%)	
**Fever**	Yes	72 (69.9%)	40 (72.7%)	32 (66.7%)	0.504
	No	31 (30.1%)	15 (27.3%)	16 (33.3%)	
**Antimicrobial resistance**					
**Amoxicillin-clavulanic**		59 (57.3%)	35 (63.6%)	24 (50%)	0.215
**Cefoxitin**		24 (23.3%)	14 (25.5%)	10 (20.8%)	0.855
**Ceftriaxone**		31 (30.1%)	21 (38.2%)	10 (20.8%)	0.015*
**Cefotaxime**		35 (34%)	27 (49.1%)	8 (16.7%)	0.001*
**Ceftazidime**		57 (55.3%)	37 (67.3%)	20 (41.7%)	0.004*
**Azetronam**		32 (31.1%)	22 (40%)	10 (20.8%)	0.065
**Imipenem**		48 (46.6%)	28 (50.9%)	20 (41.7%)	0.460
**Ciprofloxacin**		21 (20.4%)	11 (20%)	10 (20.8%)	0.926
**SXT**		83 (80.6%)	41 (74.5%)	42 (87.5%)	0.169
**Amikacin**		14 (13.6%)	6 (10.9%)	8 (16.7%)	0.2
**Gentamicin**		8 (7.8%)	4 (7.3%)	4 (8.3%)	0.841
**SXT-MIC**		73 (70.9%)	39 (70.9%)	34 (70.8%)	0.993
**ESBL phenotypic detection**					
**Double disc synergy test**		39 (37.9%)	33 (60%)	6 (12.5%)	0.000*
**Molecular Detection of resistance genes and integrons**					
***sul1***		62 (60.2%)	34 (61.8%)	28 (58.3%)	0.719
***sul* 2**		67 (65%)	37 (67.3%)	30 (62.5%)	0.612
***bla***_**TEM**_		11 (10.7%)	11(20%)	0 (0%)	0.001*
***bla***_**SHV**_		2 (1.9%)	2 (3.6%)	0 (0%)	0.182
***bla***_**CTXM9**_		6 (5.8%)	6 (10.9%)	0 (0%)	0.018*
***intI*1**		95 (92.2%)	53 (96.4%)	42 (87.5%)	0.094
***intI*2**		71 (68.9%)	37 (67.3%)	34 (70.8%)	0.697

^*^ (significant *P* value)

### Antimicrobial susceptibility of Proteus isolates

Antimicrobial susceptibility testing of the 103 *Proteus* isolates was done for 11 antibiotics belonging to eight different antimicrobial categories using the disk diffusion method. The highest antimicrobial resistance was observed against the SXT (80.6%), then AMC (57.3%), ceftazidime (55.3%), and imipenem (46.6%) antibiotics. The high susceptibility was to gentamycin, amikacin, and cefoxitin (Fig. 2 s Supplementary File). *Proteus* isolates from catheterized patients showed higher resistance to most tested antibiotics than *Proteus* isolates from non-catheterized patients; with significant differences for cefotaxime, ceftazidime, and ceftriaxone (P values were 0.001, 0.004, and 0.015, respectively); Table 1. Also, *Proteus* strains isolated from inpatients were more resistant to third-generation cephalosporins, cefoxitin, imipenem, aztreonam, amikacin, and gentamicin than those isolated from outpatients. Among 83 SXT-resistant *Proteus* isolates by the disc diffusion method, only 73 isolates (70.9%) were found to be resistant to SXT by the broth microdilution method. There was no significant difference in resistance to SXT among isolates from catheterized and non-catheterized patients; Table 1.

A high frequency of MDR was observed among all the studied isolates. Out of 103 *Proteus* strains, 81 (78.6%) were MDR. It was found that 39 MDR isolates were isolated from catheterized patients, representing 70.9% of all catheterized patients. Also, 74.6% (59/79) of *Proteus* isolates from inpatients were MDR. The 81 MDR *Proteus* isolates showed 28 antibiotic resistance profiles to 3–11 antibiotics, with MARI ranging from 0.27 to 1. Eight isolates were resistant to all tested antibiotics. MDR *Proteus* isolates were classified according to the number of resistant antimicrobial categories into five groups (Fig. 3 s Supplementary File).

### ESBL phenotypic confirmation test

A double disc synergy test was done on 43 *Proteus* strains, suspected to be ESBL producers by the disc diffusion method. Out of the suspected ESBL producer isolates, 39 stains were confirmed as a phenotypic indication of ESBL production by DDST. From the total studied 103 *Proteus* strains, there were 39 ESBL producers (37.9%) and 64 non-ESBL producers (62.1%). Thirty-three ESBL-producing *Proteus* isolates (84.6%) were from catheterized patients, and only six ESBL-producing *Proteus* isolates (15.4%) were from non-catheterized patients with a significant difference (P value 0.0001*); Table 1. All 39 ESBL-producing *Proteus* isolates were MDR. The majority of ESBL-producing *Proteus* isolates were isolated from the ICU (15 isolates), Gynaecology and Obstetrics Departments (12 isolates), and Urology Department (8 isolates). There were significant differences between the ESBL-producers and ESBL non-producers isolates in terms of the antibiotic resistance rates of imipenem, aztreonam, cefoxitin, and gentamycin antibiotics (P value: 0.007, < 0.001, 0.025, and 0.024, respectively); Table 3 s Supplementary File.

### Detection of sulphonamide resistance genes

All 103 *Proteus* isolates (73 isolates phenotypically SXT resistant + 30 isolates as control samples) were molecularly tested for the presence of the following sulphonamide-resistant genes: *sul1* and *sul2* genes. *Sul* genes were detected in 77 isolates (74.7%); with no significant differences between catheterized and non-catheterized patients; Table 1. All isolates were identified as *P. mirabilis*. Six strains harboured the *sul1* gene only, and thirteen strains harboured the *sul2* gene only. There were 58 strains harboured both *sul1* and *sul2* genes. All 73 phenotypically SXT-resistant isolates carried *sul* genes. However, four isolates had the *sul II* gene but didn't show SXT resistance by the broth microdilution method.

### Detection of ESBL genes

All 39 ESBL-producing *Proteus* isolates (*P. mirabilis*) were molecularly tested for ESBL genes *bla*_TEM_, *bla*_SHV_*, bla*_CTX-M-1, 2, 8, 9, and 25_ genes. Out of 39 isolates, 15 (38.5%) carried ESBL genes. Eleven isolates had the *bla*_TEM_ gene (28.2% of ESBL producers), and only two isolates had the *bla*_SHV_ gene (5.1%). *Bla*_CTX-M-9_ was the only detected CTX-M gene in six isolates (15.4%, two isolates harbored *bla*_CTX-M-9_ alone and 4 carried it with *bla*_TEM_), but *bla*_CTX-M 1, 2, 8, and 25_ were not detected in any isolate. All the isolates carrying ESBL genes were from catheterized patients and were all *P. mirabilis* type (Table 1). The antibiotic resistance profile of ESBL gene-producing isolates showed differences in resistance among isolates with one or two ESBL genes. There were differences in the inhibition zones in isolates with 2 ESBL genes than those with one ESBL gene. Table 4 s in the supplementary file.

### Detection of class 1 and 2 integrons

All 103 *Proteus* isolates were screened for *intI1* and *intI2* genes to detect class 1 and 2 integrons, respectively. Ninety-five isolates (92.2%) were positive for the *intI1* gene which was more frequently detected in catheterized patients (53/55; 96.4%) than in non-catheterized patients (42/48; 87.5%). The *intI2* gene was less frequently detected in *Proteus* isolates (71/103; 68.9%) with a slightly lower prevalence in catheterized (37/55; 67.3%) than in non-catheterized patients (34/48; 70.8%); Table 1. Sixty-seven *Proteus* isolates harboured both *intI1* and *intI2* genes, 28 isolates harboured the *intI1* gene only, and 4 isolates harboured the *intI2* gene only. Different seven gene profiles of ESBL, *sul,* and integron gene distribution were observed among positive ESBL gene *P. mirabilis* isolates. Four isolates carried 2 ESBL genes (*bla*_TEM_ and *bla*_CTX-M-9_). All ESBL isolates harboured *sul1* and *intI1* genes. (Table 2).

**Table 2:** Genotypic profile in 15 ESBL gene-producing *P. mirabilis* isolates

Number of isolates (15)	ESBL genes			*sul* genes		Integron genes	
	*bla*_TEM_	*bla*_SHV_	*bla*_CTX-M-9_	*Sul1*	*Sul2*	*intI1*	*intI2*
**2**	+	ND	ND	+	+	+	ND
**2**	+	ND	ND	+	+	+	+
**3**	+	ND	ND	ND	+	+	+
**2**	+	ND	+	ND	+	+	ND
**2**	+	ND	+	+	+	+	ND
**2**	ND	+	ND	+	+	+	+
**2**	ND	ND	+	+	+	+	ND

(+ gene detected; ND gene not detected)

### DNA fingerprinting analysis by ERIC-PCR

The fingerprints obtained from a PCR assay with ERIC primers of the ESBL-producing *P. mirabilis* isolates from catheterized patients (33 isolates) showed a DNA banding profile consisting of amplified bands from 100 to 3000 bp in size. The isolates had different gene profiles, and all isolates carried the intI1 gene. Table 5 s in supplementary file A dendrogram was constructed according to the data of these isolates. The isolates showed a moderate degree of similarity. The overall similarity among the 33 isolates was 65%. The isolates with the same genotypic pattern showed some similarity in ERIC patterns. (Fig. 1).

## Discussion

In recent years, *Proteus* bacteria have acquired considerable importance as causative pathogens associated with complicated UTIs. This study indicated that the rate of *Proteus* isolates was 20% among the UTI agents, with a significantly higher prevalence among CAUTIs (28.2%) than non-CAUTIs (14.9%). In the same locality; a previous study by Kotb et al.; 2019 [27], detected a lower prevalence of *Proteus* among UTI patients (4.5%) which is alarming for the dissemination of *Proteus species* within a few years, especially in hospital settings among CAUTIs which give rise to a serious health problem. In recent studies in Egypt, a similar frequency of *Proteus* among urine samples was reported [9, 28] as well as in Iraq [29]. On the other hand, Mohsin and Al-rubaii from Iraq isolated *P. mirabilis* from 29.4% of urine samples [30], which is slightly higher than our results while a lower prevalence (9.2%) was reported in Iran [8]. This different incidence of *Proteus* sp. could be attributed to different geographic regions, hygiene measures, age groups of patients, and whether the studies were hospital or community-based.

In the current study, the incidence of UTIs by *Proteus* was higher among females than males, as 63 *Proteus* isolates (61.2%) were isolated from females while 40 *Proteus* isolates (38.8%) were isolated from males. These data agree with the findings obtained by most previous studies in Egypt, Iran, and Brazil [31–33]. UTIs are more common in women than in men due to the shorter length of the female urethra which reduces the distance the organism has to travel to reach the bladder. Additionally, contamination of the urinary tract with fecal flora is more frequent.

In our study, *Proteus* isolates were more prevalent among inpatients (79 isolates, 76.7%), representing hospital-acquired UTIs, compared to outpatients (24 isolates, 23.3%), representing community-acquired UTIs. This emphasizes the important role of these bacteria as a cause of hospital-acquired infections. The prevalence of *Proteus* was significantly higher in catheterized UTI patients (28.2%) than in non-catheterized patients (14.9%). Our findings agreed with previous studies in Egypt, which reported that *Proteus* was mostly isolated from catheterized patients [31, 34]; on the other hand, a lower prevalence of proteus in CAUTI was reported in Iran (35%) [32] *Proteus* infection rates are rising in catheterized patients because of biofilm formation and the presence of several virulence factors that promote adhesion, such as flagella and IgA protease. There has been considerable investigation regarding catheter coatings designed to prevent microbial colonization and biofilm formation, termed “antifouling” [5]. Catheter coatings, catheter materials, and vaccination are novel preventive strategies for CAUTI. There has been considerable effort to develop catheters either coated or impregnated with various antibiotics e.g. nitrofurazone catheters which are available commercially. Modifying the material used for the production of catheters, such as latex, polyvinyl chloride (PVC), siliconized latex and hydrogel-coated latex [5].

Different degrees of resistance to the investigated antimicrobial drugs were detected in the antimicrobial sensitivity patterns of isolated *Proteus* species. The highest rates of resistance were against the SXT (70.9%) which matches with previous studies in Egypt (81.7%) and Iran (75%) [28, 35]. However, higher rates were reported between 90 and 100% [32, 34], as well as lower rates in Brazil (21.9%) [33] and Egypt (61%) [9]. High resistance to SXT is due to the overuse of trimethoprim/sulfamethoxazole as an empiric treatment for UTI in our country.

In the current study, we recorded the resistance to third-generation cephalosporin antibiotics; ceftazidime (55.3%), cefotaxime (34%), and ceftriaxone (30.1%). Regarding ceftazidime resistance, the result was higher than in other studies [9, 28, 35]. Regarding cefotaxime resistance, the current study agreed with Shaaban et al. in Egypt (29.3%) [9]. For ceftriaxone resistance, similar results (27%) were reported [32], and very high resistance was reported (72.7%) in Bangladesh [36]. This difference in antibiotic resistance rates can be explained by the extent of use of each antibiotic and the variation of antibiotic usage policy in these countries. Resistance to 3rd generation cephalosporins was significantly higher in *Proteus* isolated from CAUTIs than non-CAUTIs as these agents are usually administrated in CAUTIs in hospital settings [37] which complicates treatments of hospital-acquired UTIs and is alarming for dissemination of ESBL-producing organisms.

The rate of resistance to carbapenem represented by imipenem was 46.6%. Once thought to be the most effective treatment for *Proteus* infections, the acquisition of genes encoding carbapenemases and β-lactamases is a leading cause of the increased resistance to carbapenems. Ciprofloxacin is a broad-spectrum synthetic antibiotic belonging to the fluoroquinolone class and is effective in the treatment of UTIs. Ciprofloxacin resistance was recorded in 20.4% of the isolates in this study. Gentamycin and amikacin are aminoglycoside antibiotics that inhibit protein synthesis. In our study, tested *Proteus* isolates showed very high susceptibility to gentamycin and amikacin, suggesting that these antibiotics can be used to treat *Proteus* infections.

A high frequency of MDR (78.6%, 81/103) was found among all the studied isolates. The high prevalence of MDR *Proteus* strains in the current study may be due to non-indicated, random, and excessive use of these antibiotics in UTI therapy. We found that 74.6% of *Proteus* isolates from inpatients were MDR. This draws attention to the fact that *Proteus* strains, particularly *P. mirabilis,* have become a threat to hospital-acquired infections. Furthermore, 39 (70.9%) of *Proteus* isolates from catheterized patients were MDR. Biofilm formation plays a crucial role in drug resistance; as urinary catheters in the initial stages are colonized by a single species, such as *Enterococcus faecalis*, *E. coli*, *S. epidermidis*, or *P. mirabilis*. Subsequently, mixed communities develop, containing organisms such as *Providencia stuartii*, *P. aeruginosa*, *Proteus mirabilis*, and *Klebsiella pneumonia* facilitating the horizontal transfer of resistance genes of antibiotics, as transmissible plasmids that carry multiple resistance genes to other drugs giving rise to multidrug-resistant strains [38]. Strict infection control measures must be established to reduce the burden of the onset of MDR, which is a matter of concern. The MARI value that was obtained varied from 0.27 to 1, indicating a considerable increase over the highest MARI value of 0.2 [9]. The high MARI values indicate the widespread usage of the tested antibiotics in our nation and point to a substantial risk of the spread of antibiotic resistance.

The prevalence of ESBL-producing *Proteus* spp. is increasing worldwide. Our study showed that 39 *Proteus* strains were phenotypically ESBL producers (37.9%) which agreed with other studies (39%) [39]. Higher rates were reported in Egypt (51.7%) and (46.6%) [9, 40] while lower rates were reported (28.3%) in Egypt [28], Korea 22.5% [41], and Iran (10%) [8]. The difference in the prevalence of ESBL-producing *Proteus* may be due to different sample sources, antibiotic overuse, and hospital cross-infection. Prevalence of ESBL-producing *Proteu*s strains was significantly higher in CAUTI (60%) than non-CAUTIs (12.5%), which agreed with Sokhn et al. [42]. All ESBL-producing *Proteus* isolates were MDR and showed higher resistance rates than non-ESBL-producing isolates. The coexistence of resistance to multiple antibiotics demonstrates the tendency for MDR to emerge among *Proteus* strains and suggests a simultaneous and ongoing transfer of resistance traits among bacterial pathogens.

In the current study, we tested 39 phenotypic ESBL-producing *Proteus* isolates (*P. mirabilis*) molecularly for ESBL genes *bla*_TEM_, *bla*_SHV_*, bla*_CTX-M 1, 2, 8, 9, and 25_. We found that 38.5% carried ESBL genes. From 15 isolates, 11 had the *bla*_TEM_ gene (28.2%). Then, *bla*_CTX-M-9_ was the only CTX gene detected in six isolates (15.4%), but *bla*_CTX-M 1, 2, 8, and 25_ were not detected in any isolate. Only two isolates had the *bla*_SHV_ gene (5.1%). A moderate correlation was found between the presence of *bla*_TEM_ and resistance to ceftriaxone and cefotaxime (r was 0.538 and 0.473, respectively). All the isolates carrying ESBL genes were of *P. mirabilis* type. Four isolates carried 2 ESBL genes (*bla*_TEM_ and *bla*_CTX-M-9_). First, *Proteus* species have been recognized to possess the *bla*_TEM_ β-lactamase gene for a long time. Tonkić and his co-authors reported that *Proteus* isolates carried the *bla*_TEM_ gene and none of the isolates had *bla*_CTX-M_ or *bla*_SHV_ genes [24]. The *bla*_CTX-M_ gene appeared to emerge in combination with the *bla*_*TEM*_ gene at the beginning and then replace the others as the dominant gene spreading. In this study, the *bla*_TEM_ gene was the most prevalent ESBL gene. This agrees with recent results from Egypt [28, 40], Nigeria [43, 44], Iran [35], Korea [41], and China. [45]

Regarding the prevalence of *bla*_CTX-M-1,2,8,9,25_ genes, only *bla*_CTX-M-9_ was detected in our study, which agreed with other studies [33, 45]. The prevalence of *bla*_*CTX-M*_ genes has been increasing in the last few years, as reported in some studies [9, 35, 44]. The *bla*_SHV_ gene was the least detected in this study (5.1%). This agrees with other studies [35, 44], while few studies have found a higher prevalence of the *bla*_SHV_ gene [9]. These differences in the distribution of ESBL genes may be due to different geographical distributions, sources of infections, and risk factors predisposing to the emergence of such genes and the spread of MDR strains.

Molecular analysis with PCR is used to amplify sulfonamide resistance genes (*sul1* and *sul2* genes). Our results showed a high prevalence of *sul* genes (*sul1*; 60.2% and *sul2*; 65%) All these isolates were identified as *P. mirabilis.* Danilo de Oliveira and his co-authors reported a high prevalence of *sul* genes among *P. mirabilis* in Brazil [33]. Local and global reports of high prevalence of *sul* genes in clinical isolates of *E. coli* were also documented [13]. This suggests that these genes are universally involved in the transmission and carriage of sulfonamide resistance among bacteria. The *sul* genes are widely distributed as they are linked to mobile genomic elements such as integrons, transposons, and insertion sequences. The *sul1* gene is a component of class I integrons, which play a major role in the expression and dispersal of “multi-drug resistance phenotypes” as they often harbor multiple gene cassettes that confer resistance to multiple antibiotics [13]. Although all phenotypically SXT-resistant isolates carried *sul* genes, we detected four isolates carrying the *sul2* gene but didn't show SXT resistance by the broth microdilution method. This can be explained by the presence of silent antibiotic-resistance genes [46].

Class 1 and 2 integrons have been disseminated among gram-negative bacteria. Horizontal gene transfer by integrons could play vital roles in the spread of the resistance traits and the occurrence of MDR. This study reported a high prevalence of class 1 integrons (92.2%) and class 2 integrons (68.9%) similar prevalence for class 1 integrons was reported in Egypt among *proteus* isolates; 80% [47] while lower rates were reported by other studies [35, 48]. Dissemination of class 1 and 2 integrons is an alarming sign of the emergence of MDR strains. Emerging MDR bacteria are now a major cause of morbidity and mortality worldwide and are posing a treatment challenge. Effective measures for the establishment and implementation of active surveillance and infection control programs with coherent antibiotic policies in hospitals and clinics to stop and control the spread of MDR and ESBLs in hospitals and communities.

ESBL-producing *Proteus* isolates from CAUTIs were typable by ERIC-PCR. ESBL-producing isolates showed a moderate degree of genetic similarity. However, a high similarity was reported among *Proteus* strains in different previous studies. [9, 48]

## Conclusions

Our study detected a high prevalence of *Proteus* mirabilis isolated from UTIs, particularly catheterized inpatients. This highlights the role of this organism as one of the major causes of CAUTIs. Multidrug resistance was reported in 78.6% of isolates. Gentamycin was the most effective antibiotic against *Proteus*, followed by amikacin, then cefoxitin. On the other hand, *Proteus* isolates showed high resistance to SXT (80.6%) with increasing dissemination of *sul* genes among *Proteus* isolates. The current study showed increased resistance to imipenem (46.6%), which forms a high risk as it is considered the most effective antibiotic against Enterobacterals. The frequency of ESBL-producing *Proteus* isolates was 37.9%. ESBL-producing isolates showed higher resistance against many antibiotics than non-ESBL isolates. This study reported the coexistence of *bla*_TEM_ genes with *bla*_CTX-M_ genes in ESBL-producing *Proteus* isolates. We recommend further studies on *Proteus* due to insufficient available data in Egypt and continuous screening for antimicrobial susceptibility patterns of *Proteus* as one of the causes of UTI to establish updated antimicrobial policies for treatment. More antibiotic resistance genes should be examined among *Proteus* to better understand their role in increased multidrug-resistant strains and limit their dissemination. Routine molecular surveillance of *Proteus* in CAUTI cases, implementation of hospital infection control measures, and re-evaluation of empiric therapy for UTIs considering MDR trends are needed.

## Supplementary Information

Supplementary Material 1.

## Data Availability

All data generated or analysed during this study are included in this article.

## Abbreviations

CAUTI: Catheter-associated urinary tract infections
CFU: Colony forming unit
CLSI: Clinical Laboratory Standard Institute guidelines
ERIC-PCR: Enterobacterial Repetitive Intergenic Consensus‑PCR
ESBLs: Extended-spectrum beta-lactamases
MARI: Multiple antibiotic resistance index
MDR: Multiple drug-resistant
MIC: Minimum inhibitory concentrations
SUL: Sulphonamide
SXT: Trimethoprim-Sulfamethoxazole
UTI: Urinary tract infection
WHO: World Health Organization

## References

[1] Adeolu M, Alnajar S, Naushad S, S Gupta R. Genome-based phylogeny and taxonomy of the 'Enterobacteriales': proposal for Enterobacterales ord. nov. divided into the families Enterobacteriaceae, Erwiniaceae fam. nov., Pectobacteriaceae fam. nov., Yersiniaceae fam. nov., Hafniaceae fam. nov., Morganellaceae fam. nov., and Budviciaceae fam. nov. Int J Syst Evol Microbiol. 2016 Dec;66(12):5575–5599. 10.1099/ijsem.0.001485. 10.1099/ijsem.0.001485 27620848

[2] Drzewiecka D. Significance and Roles of Proteus spp. Bacteria in Natural Environments Microb Ecol. 2016Nov;72(4):741–58. 10.1007/s00248-015-0720-6. 26748500 10.1007/s00248-015-0720-6 PMC5080321

[3] Mancuso G, Midiri A, Gerace E, Marra M, Zummo S, Biondo C. Urinary Tract Infections: The Current Scenario and Future Prospects. Pathogens. 2023;12(4):623. Published 2023 Apr 20. 10.3390/pathogens12040623 10.3390/pathogens12040623 PMC10145414 37111509

[4] Yuan F, Huang Z, Yang T, Wang G, Li P, Yang B, Li J. Pathogenesis of *Proteus* mirabilis in Catheter-Associated Urinary Tract Infections. Urol Int. 2021;105(5–6):354–61. 10.1159/000514097. 33691318 10.1159/000514097

[5] Werneburg GT. Catheter-Associated Urinary Tract Infections: Current Challenges and Future Prospects. Res Rep Urol. 2022; 14:109–133. Published 2022 Apr 4. 10.2147/RRU.S273663 10.2147/RRU.S273663 PMC8992741 35402319

[6] Centers for disease control and prevention. urinary tract infection (Catheter-Associated Urinary Tract Infection [CAUTI] and non-catheter-associated Urinary Tract Infection [UTI]) Events. National healthcare safety network. 2025. https://www.cdc.gov/nhsn/pdfs/pscmanual/7psccauticurrent.pdf.

[7] Uddin TM, Chakraborty AJ, Khusro A, et al. Antibiotic resistance in microbes: History, mechanisms, therapeutic strategies and future prospects. J Infect Public Health. 2021;14(12):1750–66. 10.1016/j.jiph.2021.10.020. 34756812 10.1016/j.jiph.2021.10.020

[8] Mirzaei A., Habibi M., Bouzari S., Asadi Karam M. R. Characterization of Antibiotic-Susceptibility Patterns, Virulence Factor Profiles and Clonal Relatedness in *Proteus mirabilis* Isolates from Patients with Urinary Tract Infection in Iran. Infect Drug Resist. 2019; 12:3967–3979. Published 2019 Dec 27. 10.2147/IDR.S230303 10.2147/IDR.S230303 PMC6938180 31920349

[9] Shaaban, M., Elshaer, S.L. & Abd El-Rahman, O.A. Prevalence of extended-spectrum β-lactamases, AmpC, and carbapenemases in *Proteus* mirabilis clinical isolates. BMC Microbiol. 2022; 22: 247. 10.1186/s12866-022-02662-3 10.1186/s12866-022-02662-3 PMC9552493 36221063

[10] Vaez H, Kalarestaghi H, Sahebkar A, Khademi F. Prevalence of antibiotic resistance of Proteus species in urinary tract infections in Iran: A systematic review and meta-analysis. Gene Reports. 2022;27: 101632. 10.1016/j.genrep.2022.101632.

[11] Ojdana D, Sacha P, Wieczorek P, et al. The occurrence of blaCTX-M, blaSHV, and blaTEM genes in extended-spectrum β-lactamase-positive strains of Klebsiella pneumoniae, Escherichia coli, and *Proteus* mirabilis in Poland. International Journal of Antibiotics. 2014;2014:7. 10.1155/2014/935842.935842.

[12] Sánchez-Osuna M, Cortés P, Barbé J, Erill I. Origin of the mobile di-hydropteroate synthase gene determining sulfonamide resistance in clinical isolates. Front Microbiol. 2019;9:3332. 10.3389/fmicb.2018.03332. 30687297 10.3389/fmicb.2018.03332 PMC6335563

[13] Arabi H, Pakzad I, Nasrollahi A, et al. Sulfonamide Resistance Genes (sul) M in Extended Spectrum Beta Lactamase (ESBL) and Non-ESBL Producing Escherichia coli Isolated From Iranian Hospitals. Jundishapur J Microbiol. 2015;8(7):e19961. Published 2015 Jul 25. 10.5812/jjm.19961v2 10.5812/jjm.19961v2 PMC4584071 26421132

[14] Kaushik M, Kumar S, Kapoor RK, Virdi JS, Gulati P. Integrons in Enterobacteriaceae: Diversity, distribution and epidemiology. Int J Antimicrob Agents. 2018;51:167–76. 10.1016/j.ijantimicag.2017.10.004. 29038087 10.1016/j.ijantimicag.2017.10.004

[15] Deng Y, Bao X, Ji L, Chen L, Liu J, Miao J, Chen D, Bian H, Li Y, Yu G. Resistance integrons: class 1, 2 and 3 integrons. Ann Clin Microbiol Antimicrob. 2015Dec;14:1–1. 26487554 10.1186/s12941-015-0100-6 PMC4618277

[16] Karah N, Rafei R, Elamin W, Ghazy A, Abbara A, Hamze M, Uhlin BE. Guideline for urine culture and biochemical identification of bacterial urinary pathogens in low-resource settings. Diagnostics. 2020Oct 16;10(10):832. 33081114 10.3390/diagnostics10100832 PMC7602787

[17] Tille, Patricia M. author. Bailey & Scott's diagnostic microbiology-E-Book: Elsevier Health Sciences. 2015

[18] CLSI. Performance Standards for Antimicrobial Susceptibility Testing. 33th ed. CLSI supplement M100. Wayne, PA: Clinical and Laboratory Standards Institute.2023

[19] Magiorakos AP, Srinivasan A, Carey RB, et al. Multidrug-resistant, extensively drug-resistant and pandrug-resistant bacteria: an international expert proposal for interim standard definitions for acquired resistance. Clin Microbiol Infect. 2012;18(3):268–81. 10.1111/j.1469-0691.2011.03570.x. 21793988 10.1111/j.1469-0691.2011.03570.x

[20] Krumperman PH. Multiple antibiotic resistance indexing of Escherichia coli to identify high-risk sources of fecal contamination of foods. Appl Environ Microbiol. 1983;46(1):165–70. 10.1128/aem.46.1.165-170.1983. 6351743 10.1128/aem.46.1.165-170.1983 PMC239283

[21] Dashti AA, Jadaon MM, Abdulsamad AM, Dashti HM. Heat Treatment of Bacteria: A Simple Method of DNA Extraction for Molecular Techniques. KMJ-Kuwait Medical Journal. 2009;41(2):117–22.

[22] Li Q, Sherwood JS, Logue CM. Characterization of antimicrobial resistant Escherichia coli isolated from processed bison carcasses. J Appl Microbiol. 2007;103(6):2361–9. 10.1111/j.1365-2672.2007.03470.x. 18045421 10.1111/j.1365-2672.2007.03470.x

[23] Woodford N, Fagan EJ, Ellington MJ. Multiplex PCR for rapid detection of genes encoding CTX-M extended-spectrum (beta)-lactamases. J Antimicrob Chemother. 2006;57(1):154–5. 10.1093/jac/dki412. 16284100 10.1093/jac/dki412

[24] Tonkić M, Mohar B, Šiško-Kraljević K, et al. High prevalence and molecular characterization of extended-spectrum β-lactamase-producing *Proteus mirabilis* strains in southern Croatia. J Med Microbiol. 2010;59(Pt 10):1185–90. 10.1099/jmm.0.016964-0. 20558587 10.1099/jmm.0.016964-0

[25] Dillon B, Thomas L, Mohmand G, Zelynski A, Iredell J. Multiplex PCR for screening of integrons in bacterial lysates. J Microbiol Methods. 2005;62(2):221–32. 10.1016/j.mimet.2005.02.007. 16009279 10.1016/j.mimet.2005.02.007

[26] Michelim L, Muller G, Zacaria J, Delamare AP, Costa SO, Echeverrigaray S. Comparison of PCR-based molecular markers for the characterization of *Proteus* mirabilis clinical isolates. Braz J Infect Dis. 2008;12(5):423–9. 10.1590/s1413-86702008000500014. 19219283 10.1590/s1413-86702008000500014

[27] Kotb DN, Mahdy WK, Mahmoud MS, Khairy RMM. Impact of co-existence of PMQR genes and QRDR mutations on fluoroquinolones resistance in *Enterobacteriaceae* strains isolated from community and hospital acquired UTIs. BMC Infect Dis. 2019;19(1):979. Published 2019 Nov 21. 10.1186/s12879-019-4606-y 10.1186/s12879-019-4606-y PMC6868749 31752702

[28] Salama L., Saleh H., Abdel-Rhman S., Barwa R., Hassa, R. Phenotypic and genotypic characterization of Extended Spectrum β-lactamases producing *Proteus* mirabilis isolates. Records of Pharmaceutical and Biomedical Sciences, 2021; 5 (Pharmacognosy-Microbiology): 89–99. 10.21608/rpbs.2021.81598.1107

[29] Al-obaidi S, Al-Hashimy A. Molecular Detection of (*Urec, Mrpa, Hpma*) Genes in *Proteus mirabilis* Bacteria Isolated from Patients with Urinary Tract Infection. The Egyptian Journal of Hospital Medicine. 2023;90(1):716–20. 10.21608/ejhm.2023.279843.

[30] Mohsin M. R., AL-Rubaii B. A. L. Bacterial growth and antibiotic sensitivity of *Proteus* mirabilis treated with anti-inflammatory and painkiller drugs. Biomedicine. 2023; 43(02):728–34. https://biomedicineonline.org/index.php/home/article/view/2693

[31] El-Kazzaz S. Virulence Factors Associated with Quinolone Resistance in *Proteus* Species Isolated from Patients with Urinary Tract Infection. Egyptian Journal of Medical Microbiology, 2021; 30(1): 115–123. 10.51429/EJMM30115

[32] Tabatabaei A, Ahmadi K, Shabestari AN, Khosravi N, Badamchi A. Virulence genes and antimicrobial resistance pattern in *Proteus* mirabilis strains isolated from patients attended with urinary infections to Tertiary Hospitals. Iran Afr Health Sci. 2021;21(4):1677–84. 10.4314/ahs.v21i4.22. 35283944 10.4314/ahs.v21i4.22 PMC8889823

[33] Danilo de Oliveira W, Lopes Barboza MG, Faustino G, et al. Virulence, resistance and clonality of *Proteus* mirabilis isolated from patients with community-acquired urinary tract infection (CA-UTI) in Brazil. Microb Pathog. 2021; 152:104642. 10.1016/j.micpath.2020.104642 10.1016/j.micpath.2020.104642 PMC7938216 33246088

[34] G. Ali, D., F. M. Gad, G., A. O. Ismail, O., R. Ahmed, H., A. Ibrahem, R. Prevalence and characterization of plasmid-mediated quinolone resistance genes in *Proteus* species isolated from different patients. Novel Research in Microbiology Journal, 2023; 7(3): 1966–1981. 10.21608/nrmj.2023.300633

[35] Mirzaei A, Nasr Esfahani B, Raz A, Ghanadian M, Moghim S. From the Urinary Catheter to the Prevalence of Three Classes of Integrons, *β*-Lactamase Genes, and Differences in Antimicrobial Susceptibility of *Proteus mirabilis* and Clonal Relatedness with Rep-PCR. *Biomed Res Int*. 2021; 2021:9952769. Published 2021 Jun 10. 10.1155/2021/9952769 10.1155/2021/9952769 PMC8211507 34212042

[36] Mishu NJ, Shamsuzzaman S, Khaleduzzaman H, Nabonee MA. Association between Biofilm Formation and Virulence Genes Expression and Antibiotic Resistance Pattern in Proteus mirabilis, Isolated from Patients of Dhaka Medical College Hospital. Arch Clin Biomed Res. 2022;6(3):418–34. 10.26502/acbr.50170257.

[37] D'Incau S, Atkinson A, Leitner L, Kronenberg A, Kessler TM, Marschall J. Bacterial species and antimicrobial resistance differ between catheter and non-catheter-associated urinary tract infections: Data from a national surveillance network. Antimicrob Steward Healthc Epidemiol. 2023;3(1): e55. Published 2023 Mar 20. 10.1017/ash.2022.340. 10.1017/ash.2022.340 PMC10031580 36970431

[38] Sharma S, Mohler J, Mahajan SD, Schwartz SA, Bruggemann L, Aalinkeel R. Microbial Biofilm: A Review on Formation, Infection, Antibiotic Resistance, Control Measures, and Innovative Treatment. Microorganisms. 2023;11(6):1614. Published 2023 Jun 19. 10.3390/microorganisms11061614 10.3390/microorganisms11061614 PMC10305407 37375116

[39] Priya PS, Manonmoney, Leela KV. Phenotypic Characterisation of *Proteus Species* Isolated from Different Clinical Samples with Special Reference to Antibiotic Resistance Pattern in a Tertiary Care Centre. J Clin of Diagn Res. 2022; 16(1): DC15-DC19. 10.7860/JCDR/2022/51928.15901

[40] Mohamed ES, Khairy RMM, Abdelrahim SS. Prevalence and molecular characteristics of ESBL and AmpC β -lactamase producing *Enterobacteriaceae* strains isolated from UTIs in Egypt. Antimicrob Resist Infect Control. 2020;9(1):198. Published 2020 Dec 10. 10.1186/s13756-020-00856-w 10.1186/s13756-020-00856-w PMC7727156 33303028

[41] Ahn JY, Ann HW, Jeon Y, et al. The impact of production of extended-spectrum β-lactamases on the 28-day mortality rate of patients with Proteus mirabilis bacteremia in Korea. BMC Infect Dis. 2017;17(1):327. Published 2017 May 3. 10.1186/s12879-017-2431-8 10.1186/s12879-017-2431-8 PMC5415711 28468622

[42] Sokhn ES, Salami A, El Roz A, Salloum L, Bahmad HF, Ghssein G. Antimicrobial Susceptibilities and Laboratory Profiles of Escherichia coli, Klebsiella pneumoniae, and *Proteus* mirabilis Isolates as Agents of Urinary Tract Infection in Lebanon: Paving the Way for Better Diagnostics. Med Sci (Basel). 2020;8(3):32. Published 2020 Aug 13. 10.3390/medsci8030032 10.3390/medsci8030032 PMC7565412 32823619

[43] Alabi OS, Mendonça N, Adeleke OE, da Silva GJ. Molecular screening of antibiotic-resistant determinants among multidrug-resistant clinical isolates of Proteus mirabilis from SouthWest Nigeria. Afr Health Sci. 2017;17(2):356–65. 10.4314/ahs.v17i2.9. 29062330 10.4314/ahs.v17i2.9 PMC5637020

[44] Obadire, S., Obadire, F., Mitsan, O., Odeyinka, O., Ugbomoiko, D., Ilesanmi, I., Olakunle, O. Distribution of resistance genes encoding Extended Spectrum Beta-Lactamase (ESBL) in *Proteus* mirabilis species isolated from selected Hospitals in Jigawa State, Nigeria. Microbes and Infectious Diseases, 2023; (): -. 10.21608/mid.2023.215024.1533

[45] Xiao L, Wang X, Kong N, Cao M, Zhang L, Wei Q, Liu W. Polymorphisms of Gene Cassette Promoters of the Class 1 Integron in Clinical *Proteus* Isolates. Front Microbiol. 2019Apr;24(10):790. 10.3389/fmicb.2019.00790. 10.3389/fmicb.2019.00790 PMC6491665 31068909

[46] Deekshit VK, Srikumar S. ’To be, or not to be’-The dilemma of “silent” antimicrobial resistance genes in bacteria. J Appl Microbiol. 2022;133(5):2902–14. 10.1111/jam.15738. 35882476 10.1111/jam.15738

[47] Elsheredy A, Faisal E, El Sherbini E, Attia N. Coexistence of integrons class 1 and 2 with emergence of class 3 among Proteus mirabilis clinical isolates from Alexandria. Egypt Microbes and Infectious Diseases. 2023Feb 1;4(1):182–93.

[48] Lu W, Qiu Q, Chen K, Zhao R, Li Q, Wu Q. Distribution and molecular characterization of functional class 2 integrons in clinical *Proteus* mirabilis isolates. Infection and drug resistance. 2022Jan;1:465–74. 10.2147/IDR.S347119 PMC8858760 35210790

