# Supplementary material for: Characterization of antimicrobial resistance among Proteus mirabilis isolates from catheter-associated urinary tract infections and non-catheter-associated urinary tract infections in Egypt
Source: BMC Infect Dis. 2025 May 27;25:767. doi: 10.1186/s12879-025-11118-8 (PMC12117724; doi:10.1186/s12879-025-11118-8)
Supplement: Supplementary file 1 — Supplementary Material 1. [file 12879_2025_11118_MOESM1_ESM.pdf]

Table 1s: PCR primers used in this study and their expected amplicon sizes:

Primer	Sequence	Annealing temperature	Amplicon size (bp)	Ref.
***Sul*1**	F-5´ ACGAGATTGTGCGGTTCTTC-3′<br>R- 5´ GGTTTCCGAGATGGTGATTG-3′	55 °C	347	22
***Sul*2**	F-5´ CCGTCTCGCTCGACAGTTAT-3′<br>R-5´ GTGTGTGCGGATGAAGTCAG-3′	55 °C	506	22
***bla*$_{TEM}$**	F-5'ATGAGTATTCAACATTTCCG-3'<br>R-5'CCAATGCTTAATCAGTGAGG-3'	51 °C	832	24
***Bla*$_{SHV}$**	F-5' CGCCGGGTTATTCTTATTTGTCGC-3'<br>R-5' TCTTTCCGATGCCGCCGCCAGTCA3'	55 °C	1016	24
***bla*$_{CTX-M\ 1}$**	F-5'AAA AATCACTGCGCCAGTTC -3'<br>R-5'AGC TTATTCATCGCCACGTT-3'	52 °C	415	23
***bla*$_{CTX-M\ 2}$**	F-5' CGACGCTACCCCTGCTATT-3'<br>R-5'CCAGCGTCAGATTTTTCAGG-3'		552	23
***bla*$_{CTX-M\ 8}$**	F-5'TCGCGTTAAGCGGATGATGC-3'<br>R-5'AAC CCACGATGTGGGTAGC-3'		666	23
***bla*$_{CTX-M\ 9}$**	F-5'CAAAGAGAGTGCAACGGATG-3'<br>R-5'ATTGGAAAGCGTTCATCA CC-3'		205	23
***bla*$_{CTX-M\ 25}$**	F-5'GCA CGA TGA CAT TCG GG -3'<br>R-5'AAC CCACGATGTGGGTAGC-3'		327	23
**ERIC-1**<br>**ERIC-2**	5′-ATGTAAGCTCCTGGGGATTCAC-3′<br>5′-AAGTAAGTGACTGGGGTGAGCG-3′	48 °C		26
***intI*1**	F-5′ CAGTGGACATAAGCCTGTTC-3′<br>R-5′ CCCGAGGCATAGACTGTA-3′	55 °C	160	25
***intI*2**	F-5′ GTAGCAAACGAGTGACGAAATG-3′<br>R-5′ CACGGATATGCGACAAAAAGGT-3′	55 °C	789	25

Table 2s: Demographic and clinical data among groups of UTI patients

Characteristic		Total (n = 516)		Isolated Bacterial species				P value
				Proteus (n = 103)		Other uropathogens (n = 413)		
**Age (years)**	Mean	43.1 ± 15.1		46.1 ± 15.6		42.3 ± 14.9		0.023
	Range	18-84						
**Age group N (%)**	18-59	461	89.3%	85	82.5%	376	91%	**0.012***
	≥60	55	10.7%	18	17.5%	37	9%	
**Gender**	Male	236	45.8%	40	38.8%	196	47.5%	0.116
	Female	280	54.2%	63	61.2%	217	52.5%	
**Type of visit**	Outpatient	227	44%	24	23.3%	203	49.2%	**<0.001***
	Inpatient	289	56%	79	76.7%	210	50.8%	
**Catheterization**	Yes	195	38.3%	55	53.4%	140	33.9%	**<0.001***
	No	321	61.7%	48	46.6%	273	66.1%	
**Fever**	Yes	250	48.4%	72	69.9%	178	43.1%	**<0.001***
	No	266	51.6%	31	30.1%	235	56.9%	
**Dysuria**	Yes	382	74%	60	58.3%	322	78.0%	**<0.001***
	No	134	26%	43	41.7%	91	22.0%	

* (significant P value)

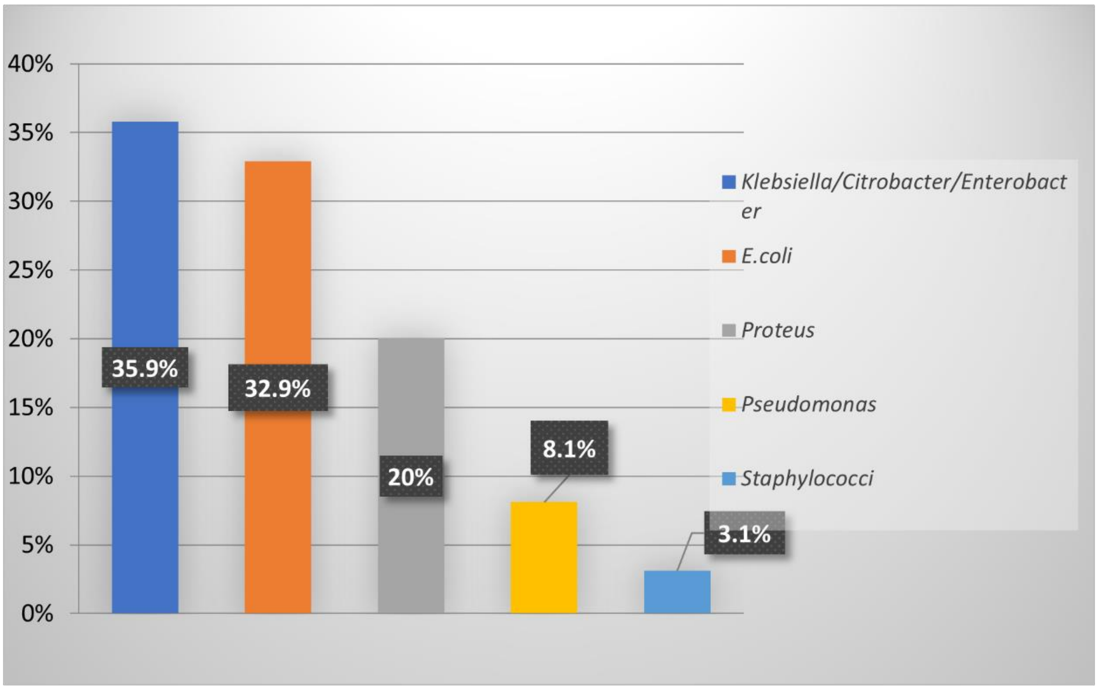

Figure 1s: Frequency of different uropathogens in urine samples

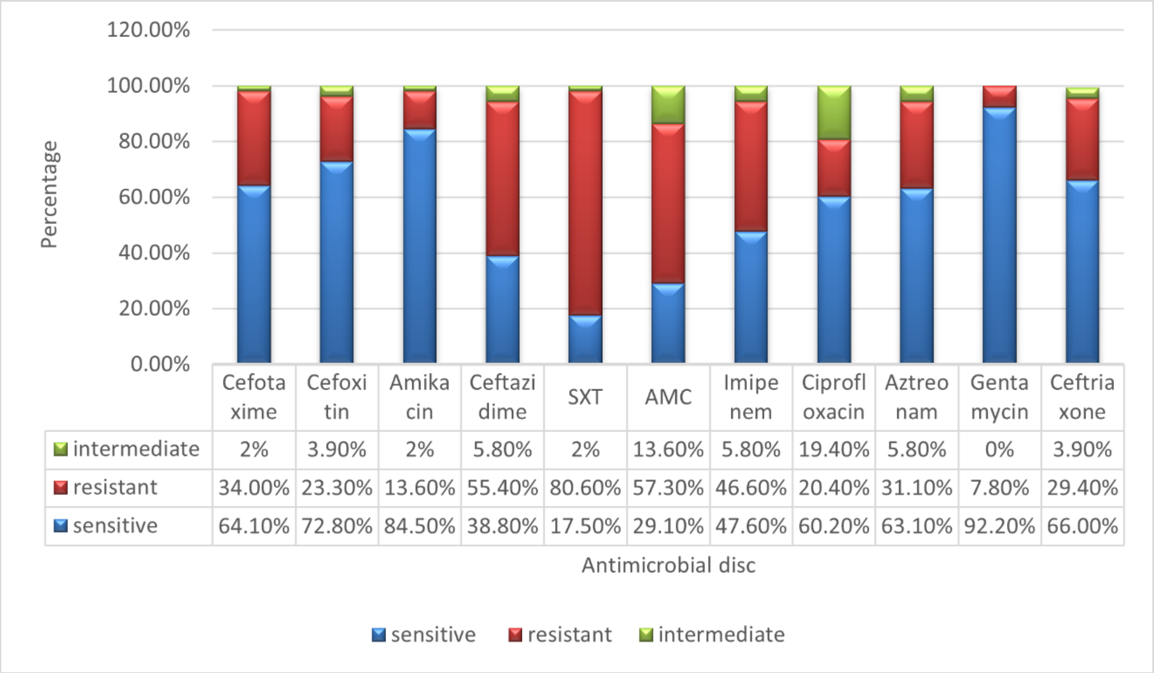

Figure 2s: Antimicrobial susceptibility patterns for isolated *Proteus* strains.

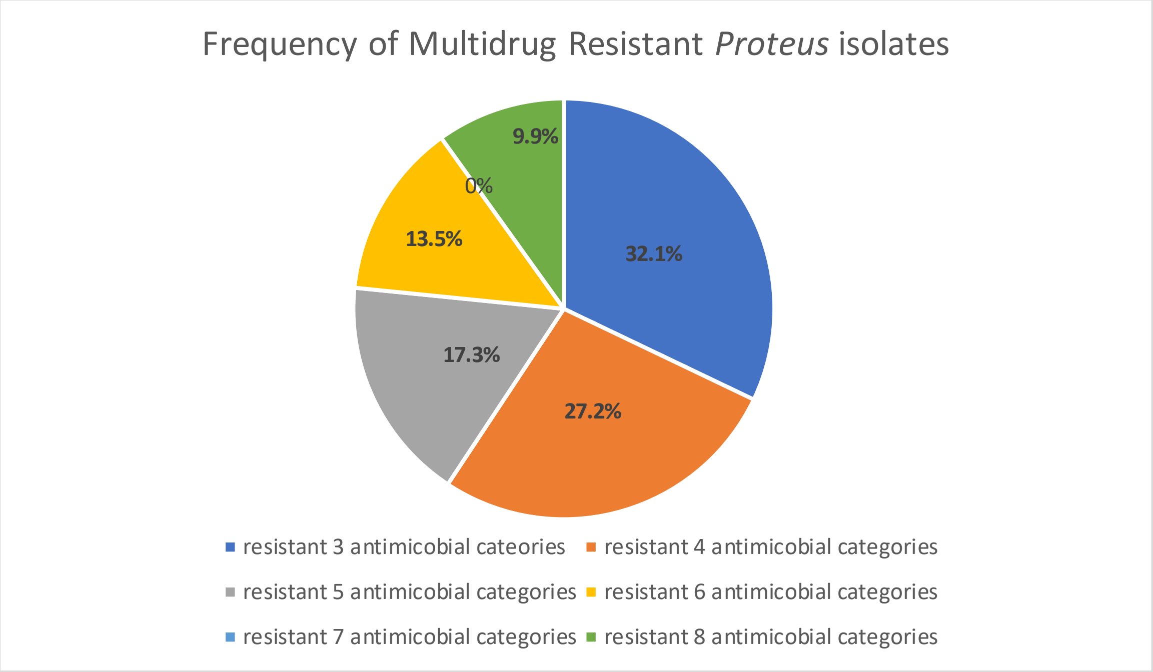

Figure 3s: Frequency of multidrug-resistant *Proteus* isolates according to the number of resistant antimicrobial categories

Table 3s: Percentage of different antibiotic resistance among ESBL-producing and non-ESBL-producing *Proteus* isolates:

Antimicrobial agent	ESBL producing isolates N=39	Non-ESBL-producing isolates N=64	P Value
**Cefoxitin**	14 (35.9%)	10 (15.6%)	**0.025***
**Amikacin**	8 (20.5%)	6 (9.4%)	0.164
**SXT**	33 (84.6%)	50 (78.1%)	0.071
**Imipenem**	24 (61.5%)	24 (37.5%)	**0.007***
**Ciprofloxacin**	11 (28.2%)	10 (15.6%)	0.279
**Aztreonam**	20 (51.3%)	12 (18.8%)	**<0.001***
**Gentamycin**	6 (15.4%)	2 (3.1%)	**0.024***

* (significant P value)

Table 4s: antibiotic resistance profiles of the 15 ESBL gene-producing *P. mirabilis*

ESBL Isolate	IMP	CIP	CN	FOX	AK	SXT	*sul1*	*sul2*	ATM	AMC	CTX	CAZ	CRO	*bla*$_{TEM}$	*bla*$_{SHV}$	*bla*$_{CTX-M9}$	*intI1*	*intI2*
1	27-S	20-R	17-S	25-S	20-S	0-R	No	yes	32-S	0-R	18-R	0-R	12-R	yes	No	No	+	+
2	25-S	10-R	20-S	9-R	25-S	0-R	No	yes	14-R	0-R	12-R	0-R	0-R	yes	No	yes	+	-
3	12-R	24-I	20-S	20-S	18-S	0-R	yes	yes	16-R	14-I	27-S	15-R	26-S	No	No	yes	+	-
4	32-S	28-S	20-S	24-S	22-S	0-R	yes	yes	30-S	0-R	0-R	0-R	0-R	yes	No	yes	+	-
5	0-R	40-S	20-S	22-S	24-S	0-R	yes	yes	30-S	20-S	12-R	0-R	12-R	yes	No	No	+	-
6	0-R	28-S	18-S	0-R	18-S	0-R	yes	yes	14-R	0-R	10-R	0-R	10-R	yes	No	No	+	+
7	0-R	26-S	22-S	0-R	18-S	0-R	yes	yes	20-I	0-R	0-R	0-R	14-R	No	yes	No	+	+
8	26-S	18-R	18-S	10-R	20-S	0-R	No	yes	12-R	0-R	0-R	0-R	12-R	yes	No	yes	+	-
9	30-S	20-R	18-S	25-S	22-S	0-R	No	yes	31-S	0-R	17-R	28-S	15-R	yes	No	No	+	+
10	10-R	23-I	19-S	20-S	25-S	0-R	yes	yes	16-R	0-R	29-S	13-R	28-S	No	No	yes	+	-
11	30-S	28-S	23-S	25-S	25-S	0-R	yes	yes	28-S	0-R	0-R	15-R	0-R	yes	No	yes	+	-
12	0-R	36-S	22-S	20-S	24-S	0-R	yes	Yes0.	30-S	22-S	15-R	30-S	14-R	yes	No	No	+	-
13	12-R	27-S	23-S	0-R	20-S	0-R	yes	yes	22-I	0-R	10-R	14-R	13-R	No	yes	No	+	+
14	28-S	33-S	22-S	22-S	18-S	0-R	yes	yes	16-R	0-R	13-R	0-R	0-R	yes	No	No	+	+
15	28-S	10-R	19-S	25-S	22-S	0-R	No	yes	30-S	10-R	15-R	0-R	25-S	yes	No	No	+	+

Table 5s: Different gene profiles of the phenotypic ESBL-producing *P. mirabilis* isolates from catheterized patients

Number of isolates (33)		*sul* genes		ESBL genes			Integron genes	
		Sul1	*Sul2*	*bla*$_{TEM}$	*bla*$_{SHV}$	*bla*$_{CTX-M-9}$	*intI1*	*intI2*
ESBL gene producers	2	+	+	+	ND	ND	+	ND
	2	+	+	+	ND	ND	+	+
	3	ND	+	+	ND	ND	+	+
	2	ND	+	+	ND	+	+	ND
	2	+	+	+	ND	+	+	ND
	2	+	+	ND	+	ND	+	+
	2	+	+	ND	ND	+	+	ND
Non-ESBL gene producers	2	+	ND	ND	ND	ND	+	ND
	2	+	+	ND	ND	ND	+	+
	2	+	+	ND	ND	ND	+	ND
	4	ND	ND	ND	ND	ND	+	+
	6	+	+	ND	ND	ND	+	+
	2	ND	ND	ND	ND	ND	+	ND

(+ gene detected; ND gene not detected)
